# Standardizing the Protocol for Hemispherical Photographs: Accuracy Assessment of Binarization Algorithms

**DOI:** 10.1371/journal.pone.0111924

**Published:** 2014-11-24

**Authors:** Jonas Glatthorn, Philip Beckschäfer

**Affiliations:** 1 Department of Plant Ecology, Albrecht von Haller Institute of Plant Sciences, Georg-August-Universität Göttingen, Göttingen, Germany; 2 Chair of Forest Inventory and Remote Sensing, Georg-August-Universität Göttingen, Göttingen, Germany; Fondazione Edmund Mach, Research and Innovation Centre, Italy

## Abstract

Hemispherical photography is a well-established method to optically assess ecological parameters related to plant canopies; e.g. ground-level light regimes and the distribution of foliage within the crown space. Interpreting hemispherical photographs involves classifying pixels as either sky or vegetation. A wide range of automatic thresholding or binarization algorithms exists to classify the photographs. The variety in methodology hampers ability to compare results across studies. To identify an optimal threshold selection method, this study assessed the accuracy of seven binarization methods implemented in software currently available for the processing of hemispherical photographs. Therefore, binarizations obtained by the algorithms were compared to reference data generated through a manual binarization of a stratified random selection of pixels. This approach was adopted from the accuracy assessment of map classifications known from remote sensing studies. Percentage correct (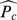) and kappa-statistics (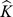) were calculated. The accuracy of the algorithms was assessed for photographs taken with automatic exposure settings (auto-exposure) and photographs taken with settings which avoid overexposure (histogram-exposure). In addition, gap fraction values derived from hemispherical photographs were compared with estimates derived from the manually classified reference pixels. All tested algorithms were shown to be sensitive to overexposure. Three of the algorithms showed an accuracy which was high enough to be recommended for the processing of histogram-exposed hemispherical photographs: “Minimum” (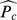 98.8%; 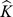 0.952), “Edge Detection” (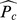 98.1%; 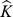 0.950), and “Minimum Histogram” (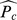 98.1%; 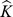 0.947). The Minimum algorithm overestimated gap fraction least of all (11%). The overestimation by the algorithms Edge Detection (63%) and Minimum Histogram (67%) were considerably larger. For the remaining four evaluated algorithms (IsoData, Maximum Entropy, MinError, and Otsu) an incompatibility with photographs containing overexposed pixels was detected. When applied to histogram-exposed photographs, these algorithms overestimated the gap fraction by at least 180%.

## Introduction

Hemispherical photography is an important and frequently applied technique to assess light conditions and canopy structure in forests [1]. Information about available radiation, as derived from hemispherical photographs, e.g. allows for investigating light response of natural regeneration or habitat choice by insects [2]. This information can also be used to model tree growth in forest ecosystems, e.g. with the software BWINPro [3]. The techniques major drawback is that obtained values are often not comparable among studies due to non-standardized exposure determination procedures applied during the acquisition [4],[5] and non-standardized binarization methods applied in the processing of hemispherical photographs [6],[7]. The impact of exposure determination methods on hemispherical photographs was e.g. investigated by [4] [8] [9] and [5]. For dark canopy conditions [5] found that gap fraction values can be up to 900% higher if photographs were auto-exposed and not non-overexposed as recommended by e.g. [9]. In the present study we assumed that non-overexposed photographs can be binarized with a higher accuracy than auto-exposed photographs. Nevertheless, auto-exposed photographs were included in the study because auto-exposure is still an often applied exposure determination method in hemispherical photography [5].

In processing hemispherical photographs the binarization or so-called “thresholding” which classifies all pixels of a photograph into either two or three categories is commonly the first step:

- Most software classify all pixels in a photograph into two categories: sky and vegetation (binarization, Table 1; [6]). Therefore, a global threshold is determined and all pixels with a gray value below or equal that threshold are classified as vegetation, remaining pixels are classified as sky. The threshold is either determined subjectively by an operator (interactive thresholding - e.g. GapLightAnalyzer, Forest Renewal BC, [10]) or automatically by an algorithm (e.g. Win-SCANOPY, Régent Instruments, Canada). Some software additionally allow for an independent threshold determination for separate sectors of the photograph (local thresholding - e.g. CAN-EYE, INRA 2010).

**Table 1:** Binarization methods implemented in currently available software for the processing of digital hemispherical photographs.

Software	Binarization method	Level	Included in this study	Literature related to the binarization	Distribution
CAN-EYE	True color	Pixel or sub-pixel	No	Not described	Freeware (INRA 2010)
DHP.exe	Interactive global or local thresholds	Sub-pixel	-	-	Freeware (Leblanc et al 2005)
Gap Light Analyzer (GLA)	Interactive threshold	Pixel	No	-	Freeware (Frazer et al. 1999)
Hemisfer	Automatic global or local threshold (2 different)	Pixel	Yes	Nobis and Hunziker 2005, Ridler and Calvard 1978	Proprietary (Schleppi)
Hemiview	Interactive global threshold	Pixel	No	-	Proprietary, Delta-D Devices, Cambridge, UK
LIA32	Automatic global threshold (3 different)	Pixel	Yes	Otsu 1979, Kittler and Illingworth 1986, Kapur et al. 1985	Freeware (Yamamoto 2004)
RGB-Fisheye	Interactive or automatic global threshold	Pixel	No	Ishida 2004	Freeware (Ishida 2005)
SideLook (binary-zation only)	Automatic global threshold	Pixel	Yes	Nobis and Hunziker 2005	Proprietary (Nobis2005)
Winphot	Interactive global threshold	Pixel	-	-	Freeware (ter Steege)
Win-SCANOPY	Classification based on true color; automatic global threshold	Pixel	No	Not described	Proprietary, Régent Instruments, Canada

- Besides the classes vegetation and sky, mixed pixels are also distinguished i.e. pixels covered by both vegetation and sky. For mixed pixels the fractions of their represented solid angle of the hemisphere covered by vegetation is calculated on a sub-pixel level. Variants of this method are e.g. suggested by [9],[11],[12].

The multitude of available software products and thresholding algorithms questions the comparability of results obtained by different studies and, in consequence, urges for a standardization of the approach. [6],[13], and [14] are among others studies dealing with this issue. [6] quantified the accuracies of a wide range of automatic global thresholding algorithms. They compared binarized hemispherical photographs against photographs that were interactively binarized by an operator; accuracies were described with the method by [15]. The IsoData algorithm [16] was proposed to be the optimal thresholding algorithm for processing hemispherical photographs.

[13] investigated how binarization algorithms impacted on indices derived from hemispherical photographs, e.g. leaf area index (LAI) and canopy openness. [13] found significant differences but concluded that they were not substantial and had little impact on the results.

[14] analyzed the thresholding algorithms Maximum Entropy [17], MinError [18], and Otsu's method [19] implemented in the software LIA32 [20]. As a reference, hemispherical photographs were interactively binarized by 21 operators. The median of the manually defined thresholds was assumed to be optimal and was used for the evaluation of the thresholding algorithms. The algorithm Maximum Entropy was found to be biased towards lower threshold values and larger gap fractions. Otsu's method was proposed to be the best algorithm for photographs with a low gap fractions (<10%), MinError was judged to be appropriate for photographs with higher gap fractions.

[21] introduced the linear ratio method which circumvents the binarization of hemispherical photographs to estimate canopy gap fraction. It requires that photographs taken beneath the canopy are related to photographs taken simultaneously above the canopy or on close-by open land locations. Using plant area and clumping index values estimated with the LAI 2000 Plant Canopy Analyzer (LI-COR, Lincoln, Nebraska, USA) as a reference, [21] compared the results of the linear ratio method with those obtained from photographs which were either thresholded interactively or by the algorithms IsoData [16] or Edge Detection [22]. [21] concluded that the linear ratio method was of higher accuracy than the other techniques. But as the linear ratio method requires taking reference photographs it is more time consuming.

In the assessment of accuracies, subjectivity becomes an important issue as soon as a human operator is involved in the classification process [6],[23]. To circumvent subjectivity, several studies assessed the accuracy of binarization algorithms by comparing specific parameters derived from hemispherical photographs (e.g. LAI) to values acquired with ”non-photographic” methods (e.g. LAI-2000; LI-COR, Lincoln, Nebraska, USA) [13],[21],[22],[24],[25]. This approach allows for the exclusion of subjective steps during the evaluation process, but has drawbacks: (1) results are influenced by possible mistakes made by the devices used as a reference, see [26] for the LAI-2000, (2) classification errors which compensate for each other cannot be detected, and (3) there is no consensus in the scientific community on how to derive LAI values from hemispherical photographs [6]. Another approach to evaluate classification algorithms is to compare automatically thresholded photographs against those interactively thresholded by human operators (e.g. [6],[14]); also this approach suffers from subjectivity. A comparison of 10 photographs, thresholded by 10 different operators was done by [6] to assess the operators' impact on gap fraction values. They concluded that the operator dependent thresholding provides a disturbing factor that interferes with reproducibility, and hampers a reliable comparison of sites and studies.

To reduce subjectivity, for the acquisition of reference data we applied an approach frequently used in the accuracy assessment of remote sensing products [27] and studies on character recognition [28]. Within each photograph single pixels were selected and assigned to the classes sky or vegetation by the author. On a pixel basis this discrimination can be made very accurately, especially if compared to setting a global threshold which requires the operator to pay attention to all parts of the photograph simultaneously. Nevertheless, a human operator is involved, and therefore, also this approach is to a certain degree inherently subjective [28]. However, it is a widely applied method to assess the accuracy of binarization algorithms for document images [29] and it is assumed to provide satisfactory results for hemispherical photographs as well.

Seven binarization algorithms implemented in publicly available software or recommended for the processing of hemispherical photographs in the literature were evaluated (Table 1). Based on objective measures of binarization accuracy, algorithms which ensure the best possible output were identified.

## Methods

### Acquisition of hemispherical photographs

Two hemispherical photographs with different exposure settings were taken at ten locations along a gradient of canopy closure in Xishuangbanna Tropical Botanical Garden, Yunnan, China (UTM/WGS 84: 47N 732940 E, 2426540 N). No specific permissions were required for field studies and no endangered or protected species were involved. All photographs and data is available from the Dryad Digital Repository: http://doi.org/10.5061/dryad.s9652.

A Nikon D70s DSLR camera equipped with a Sigma Circular Fisheye 4.5 mm 1∶2.8 lens with a field of view of 180° was used. The camera was mounted on a tripod at 1.2 m height to characterize the canopy without the interfering presence of understory vegetation [30]. The camera was leveled to face exactly the vertical using a bubble-level. The top of the camera (position of the flash socket) was orientated to magnetic north using a compass [31]. Photographs were taken without direct sunlight entering the lens [32] in the early morning, late afternoon, or on overcast days as suggested by [33].

At each location, one photograph was taken with the camera settings mode “P” (Programmed Auto), ISO  = 400, and matrix metering. By using the exposure compensation function of the camera (+-EV), photographic exposure was determined following the histogram-exposure protocol [5] which exposes the photograph to the brightest spot within the scene, i.e. the sky, and thus, prevents overexposure. A second photograph was taken at each location using the auto-exposure mode of the camera.

**Figure 1:**
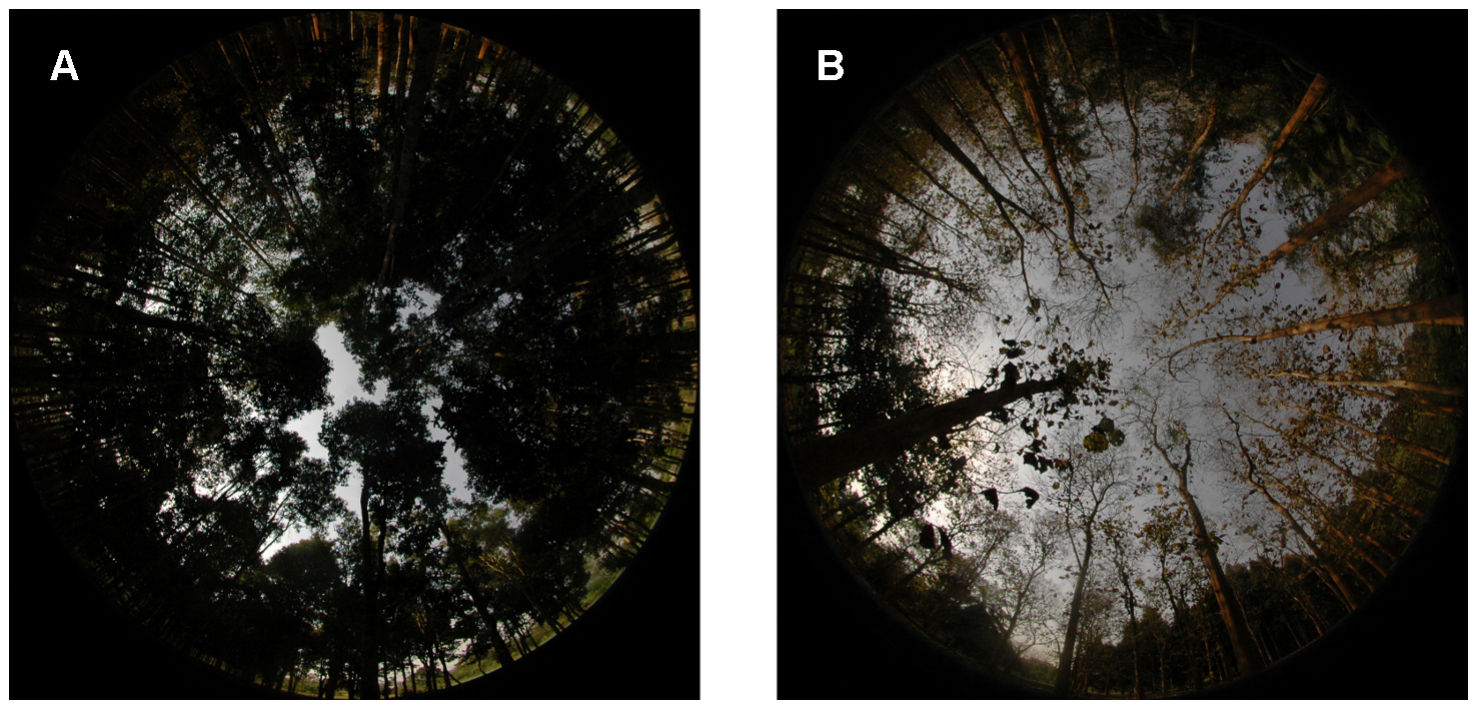
Hemispherical photographs with high and low amounts of mixed pixels. Histogram-exposed photographs of site V (A) with foliated vegetation and a low amount of mixed pixels and site VIII (B) with defoliated vegetation and a high amount of mixed pixels.

Visual inspection of the photographs revealed that the vegetation in locations VIII and IX was, in contrast to that in the other photographs, not foliated, and therefore, mainly composed of fine structures i.e. small branches and twigs (Figure 1). These fine structures resulted in a large amount of mixed pixels. Since mixed pixels are influenced by sky and vegetation likewise, it is difficult to determine to which class they eventually belong to. The results of these photographs were presented but excluded from the statistical analyses.

### The binarization algorithms

The photographs were classified into sky and vegetation through the application of the algorithms to the photographs' blue color planes. This was expected to provide the best results because of a high contrast between vegetation and sky resulting from low scattering of blue light by leaves [21]. All algorithms determined a global threshold for binarization. As some software (WinSCANOPY, CAN-EYE; Table 1) only store binarized photographs internally but do not offer the option to export them, we were not able to include the binarization methods of these software in the analyses. Methods which classify pixels in more than two categories (e.g. [12]) or circumvent pixel classification [21] were not included either.

The following seven algorithms were evaluated in this study; if not stated otherwise the algorithms are implemented in the Auto Threshold Plugin in ImageJ [34]:

- **Edge Detection** [22] implemented in SideLook [35]: For each possible threshold the contrast of neighboring pixels classified as vegetation and sky is quantified (Edge Value). The threshold with the maximal Edge Value is used for the binarization.
- **IsoData** [16]: The class mean levels of the probability distributions of both classes (vegetation and sky) are iteratively calculated for increasing thresholds. The first threshold which separates the difference of the class mean levels of both distributions in two equally large sections is applied to the photograph.
- **Maximum Entropy** [14] in [17]: The gray value histogram is divided by a possible threshold in two separate probability distributions for vegetation and sky pixels. Subsequent, for each of the distributions the entropies are calculated. The threshold which maximizes the sum of the entropies is chosen.
- **MinError** [18]: It is assumed that the threshold divides the gray value histogram in two normally distributed populations. With the Bayes formula an average classification error for each possible threshold, dependent on the class mean levels and the class variances, is calculated. The threshold with the minimum error is used for the classification.
- **Minimum** [36]: The gray value histogram is iteratively smoothed through repeated application of a moving average over three neighboring gray values. As soon as a single minimum between two modes is reached in a histogram, the minimum bin of this histogram is used as threshold.
- **Minimum Histogram** [5]: This method iteratively calculates new gray value histograms with increasing bin widths. The gray value of the left border of the first bin is always zero. As soon as exactly one minimum between two modes exists in a histogram, the optimal threshold is defined as the middle of this minimum bin of the histogram. An in-house developed R-script is used for its calculation.
- **Otsu** [19]: For both classes (vegetation and sky) the probabilities of class occurrence and class mean levels are calculated for each possible threshold. The threshold with the maximum between-class variance is used.

### Accuracy assessment of the binarization algorithms

**Figure 2:**
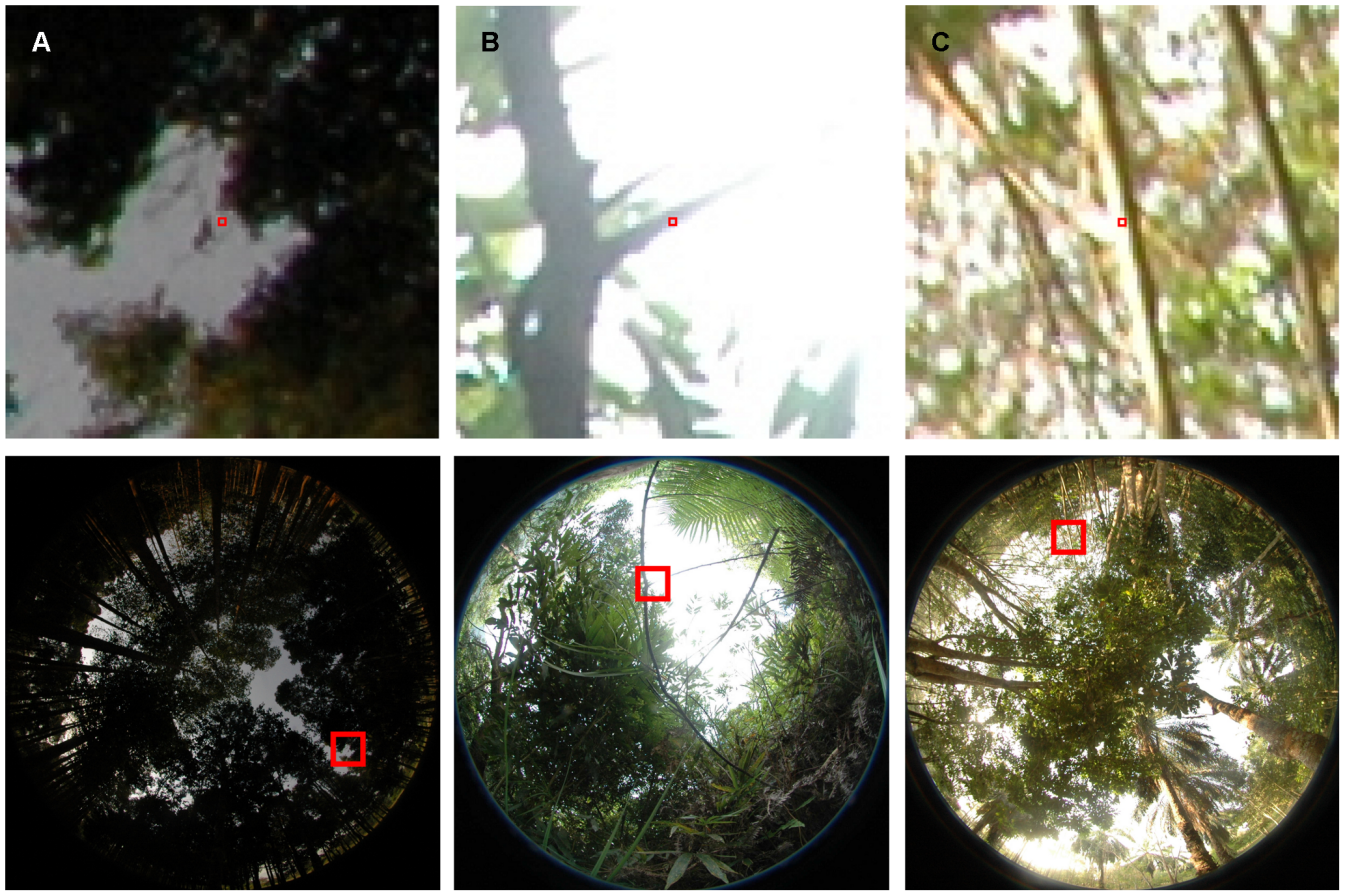
Examples of reference pixels. (A) Pixel with a low gray value in a dark region of the sky; (B) blooming effect (overexposed vegetation pixel); (C) reflection at vegetation.

The accuracies of the binarization algorithms were quantified using percentage correct [27] and the kappa-statistic (originally suggested by [37]). Both statistics are standard measures in remote sensing studies to assess the accuracies of image classifications. To calculate the two statistics a reference, usually generated based on expert opinion and considered true is required [28] [38]. In this study, reference data were obtained through the manual binarization of *n* = 384 pixels sampled from each photograph. To ensure that reference pixels were distributed across the whole range of possible gray values, each photograph was stratified into *h* = 16 strata, covering a range of 16 gray values respectively. The sample size per stratum was 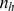  = 24 pixels. Selected pixels were classified manually into vegetation and sky on single pixel basis. For this purpose the sections of the photographs surrounding a reference pixel were magnified and examined (Figure 2). Since the gray value of a pixel within a hemispherical photograph is not only influenced by its origin (sky or vegetation) the following additional aspects were taken into account to classify the pixels:

- **Position of a pixel within the photograph**: The illumination changes across the different sections of a photograph (Figure 2 A); therefore, pixels close to the zenith are brighter than pixels close to the horizon [39].
- **Blooming effect**: Saturated sky pixels influence the gray value of neighboring vegetation pixels (Figure 2 B) [11].
- **Reflection at vegetation**: The angle of incidence of sunlight to a surface or a light color of a leaf may lead to a higher gray value than is the case for the rest of the vegetation (Figure 2 C).

### Accuracy assessment of the classification algorithms

#### Confusion matrix

**Figure 3:**
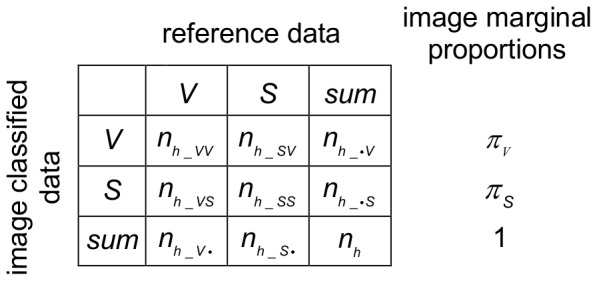
Confusion matrix. The matrix is calculated for the 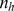  = 24 reference pixels of each of 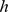  = 16 gray value-strata of a hemispherical photograph. Column and row sums show how many pixels were classified by a binarization algorithm (image classified data) and by an operator (reference data) into the categories vegetation (V) and sky (S). The four central cells show how many pixels were classified by an algorithm and the operator in agreement (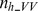, 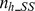) and in disagreement (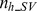, 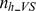). The image marginal proportion displays the overall fraction of pixels classified by the algorithm as vegetation (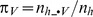) or sky (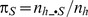) within the respective stratum. Adapted from Congalton and Green 2009.

A confusion matrix is a contingency table which displays how two classifications comply with each other [27]. In our case the matrix showed the numbers of reference pixels per stratum *h* and stated how many of them were classified correctly or not by the binarization algorithm (Figure 3). From this matrix the accuracy measures percentage correct and kappa were estimated.

#### Percentage correct

Percentage correct is defined as the fraction of pixels which was classified correctly [27]. We calculated percentage correct for each stratum 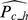, then, the overall 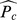 was calculated per photograph by averaging the per strata values weighted by their strata sizes: 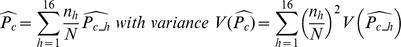
with 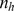 being the number of pixels of stratum *h* and *N* being the total number of pixels in the photograph. The percentage correct and its variance for the single strata were estimated using the equation:
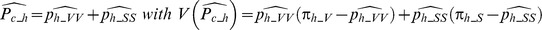
with 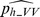 and 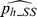 being the probabilities of a randomly selected pixel to be classified correctly as vegetation (VV) or sky (SS). The estimated probabilities were corrected for bias using the image marginal proportions 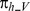 and 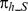:
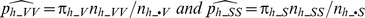



The notation of the confusion matrix shown in Figure 3 was used.

#### Kappa

The kappa-statistic [27] compares the agreement of an algorithm's classification and the reference
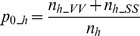
with the agreement of a random classification:



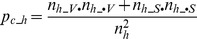
The kappa estimate per stratum was given by



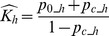
For each photograph the overall kappa was calculated as the weighted average of the per strata kappa estimates:



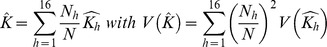



The calculation of the variance of kappa for the single strata was not formulated here (see [27] for details).

### Gap fraction

The effect of misclassification on subsequent results was demonstrated by means of the gap fraction, which is the basic measure for the calculation of several canopy structure related indices, like LAI [40] and the direct and indirect site factors [41]. Gap fraction 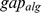 was defined as the portion of a photograph's pixels classified as sky and was calculated for the whole photograph. No separation into specific regions of the hemisphere, as e.g. in [6],[42] was done. This variant of gap fraction is sometimes also referred to as canopy openness [10] or sky-view factor [43].

Misclassification of pixels does not directly lead to a misestimation of gap fraction. If the same amount of misclassified pixels of a hemispherical photograph is classified as sky and vegetation, gap fraction does not change compared to a classification which is perfectly in agreement with the reference. To assess the actual impact of misclassifications, therefore, gap fraction was estimated on the basis of the reference pixels as well:
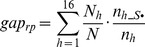
with 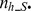 being the numbers of reference pixels classified as sky by the operator and 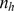 being the sample size in stratum 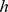. The misestimation of the gap fraction by the algorithms was calculated by dividing 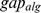
through 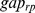.

### Data analysis

The accuracies of the different binarization algorithms were estimated and their implications for gap fraction values as derived from hemispherical photographs were assessed. Analyses of the dependence of percentage correct, kappa, and gap fraction on the algorithms were carried out for differently exposed photographs separately. The residuals of two-way ANOVA models were tested for normal distribution and sphericity (Kolmogorov-Smirnov-test and Mauchly's-sphericity-test). Based on these tests, normal distribution of kappa was accepted but percentage correct and gap fraction were not normally distributed. For transformed values (percentage correct: exponentiation by ten; gap fraction: logarithm to the basis of ten) the normal distribution was accepted. All following statistical analyses were done with the transformed values.

Sphericity had to be declined for all statistical models; therefore, the very robust Bonferroni procedure was applied [44]. Accordingly, for all multiple comparisons of the effects of the algorithms and the exposure settings t-tests with Bonferroni-adjusted p-values were used. All statistical tests were done with a significance level of 0.95 and throughout the paper the error of parameter estimates was reported with a 95% confidence interval. All statistical processing was done in R 2.15.3 [45].

## Results

### Quantitative evaluation of the classification algorithms

**Figure 4:**
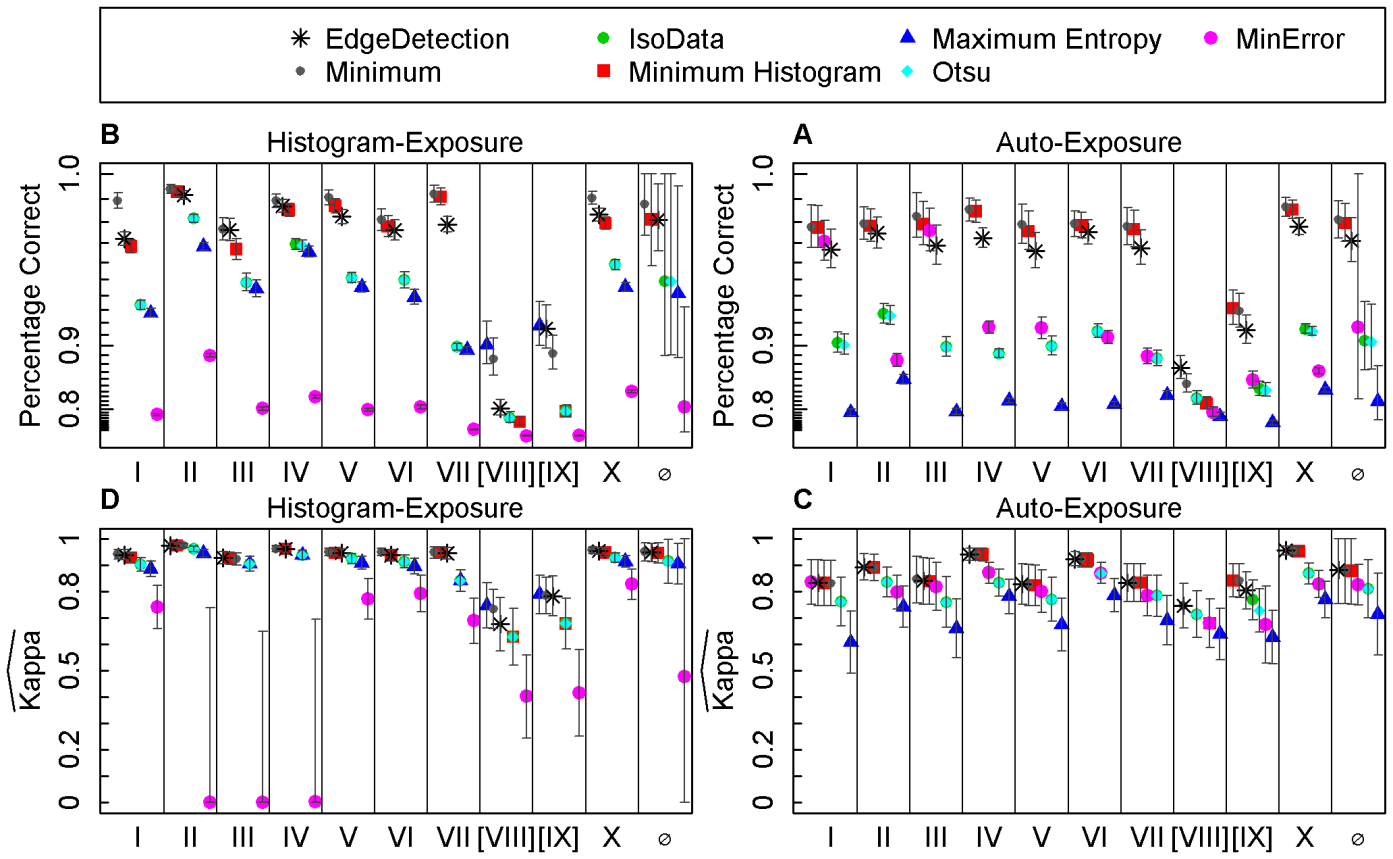
Accuracy assessment of seven binarization algorithms for hemispherical photographs . Percentage correct and kappa values of binarizations obtained from different algorithms applied to hemispherical photographs. Photographs were taken at ten locations (indicated by latin numbers) and either histogram- or auto-exposed. Whiskers represent confidence intervals with a confidence level of 95%.

Due to large amounts of mixed pixels the estimated accuracies of photographs VIII and IX differed greatly from those of the other photographs (Figure 4). Not considering these two photographs, the algorithms can be clustered by their binarization accuracies into three groups. The algorithms that ranked highest according to percentage correct and kappa were Minimum, Minimum-Histogram, and Edge Detection. All three algorithms achieved constantly high binarization accuracies for both exposure settings. The second group consisting of the algorithms IsoData, Otsu, and Maximum-Entropy had lower estimated accuracies and higher variances than those of the first group. Their estimated accuracies were higher with histogram-exposed photographs than with auto-exposed photographs. The differences of the mean accuracies of the algorithms of the first group to those of the second group were significant for all auto-exposed photographs (p-value <0.00074). For the histogram-exposed photographs most differences were significant as well. The last group consisted of the MinError algorithm only. Its accuracy was higher with auto-exposed photographs than with histogram-exposed ones; its accuracy estimates showed a high variation with some values being similar to those of the first group but very low values for some photographs as well.

The Minimum algorithm achieved independent of the exposure settings (auto-exposure: AE and histogram-exposure: HE) the highest percentage correct estimate for all analyzed photographs (AE: 98.1%±0.8%, HE: 98.8%±1.4%, Figure 4 A and B). The difference between the percentage correct estimates of the Minimum algorithm and the second and third ranked algorithms Minimum Histogram (AE: 97.9%±0.9%, HE: 98.1%±1.5%) and Edge Detection (AE: 97.1%±1.1%, HE: 98.1%±2.3%) were small (<0.97%).

The estimated kappa values of the three algorithms showed similar patterns as the percentage correct values. Except for photograph no. I, the algorithms Minimum (AE: 0.881±0.123, HE: 0,952±0.034), Minimum-Histogram (AE: 0.878±0.126, HE: 0,947±0.038), and Edge Detection (AE: 0.883±0.126, HE: 0,950±0.035) were ranked highest with minor, non-significant differences between one another (Figure 4 C and D) for both exposure settings (AE and HE). The kappa values of the classifications of auto-exposed photographs had a more than ten times higher variance than those of histogram-exposed photographs.

The algorithms Otsu and IsoData produced almost identical results. The threshold values of both were either the same or one gray value apart from each other. The percentage correct estimates of their classifications were 90.4%±0.03% (AE) and 94.9%±5.91% (HE). The kappa estimates were 0.812±0.111 (AE) and 0.916±0.083 (HE). Differences between the exposure settings were significant in both cases (p-value <0.0016). The Maximum Entropy algorithm achieved lower percentage correct (AE: 82.0%±5.8%, HE: 94.1%±5.4%) and kappa (AE: 0.715±0.155, HE: 0.906±0.076) estimates than the IsoData algorithm. The classifications of the histogram-exposed photographs of all three algorithms were equally accurate (no significant difference, p-value>0.23). The auto-exposed photographs were classified by Maximum Entropy with a significantly lower percentage correct and kappa than by IsoData and Otsu (p-value <0.0005).

The MinError algorithm classified the photographs with varying accuracies. The auto-exposed photographs were classified with a percentage correct between 87% and 98% and a kappa between 0.79 and 0.87. The histogram-exposed photographs were classified with a lower accuracy with a percentage correct between 70% and 89% and a kappa between 0.00 and 0.87. The differences of MinError to the other algorithms were significant for the percentage correct estimates of the histogram-exposed photographs (p-value <0.00052). The kappa estimates of all photographs and the percentage correct estimates of the auto-exposed photographs of MinError were not significantly different to the other algorithms.

### Impact of the binarization algorithms on gap fraction values

**Figure 5:**
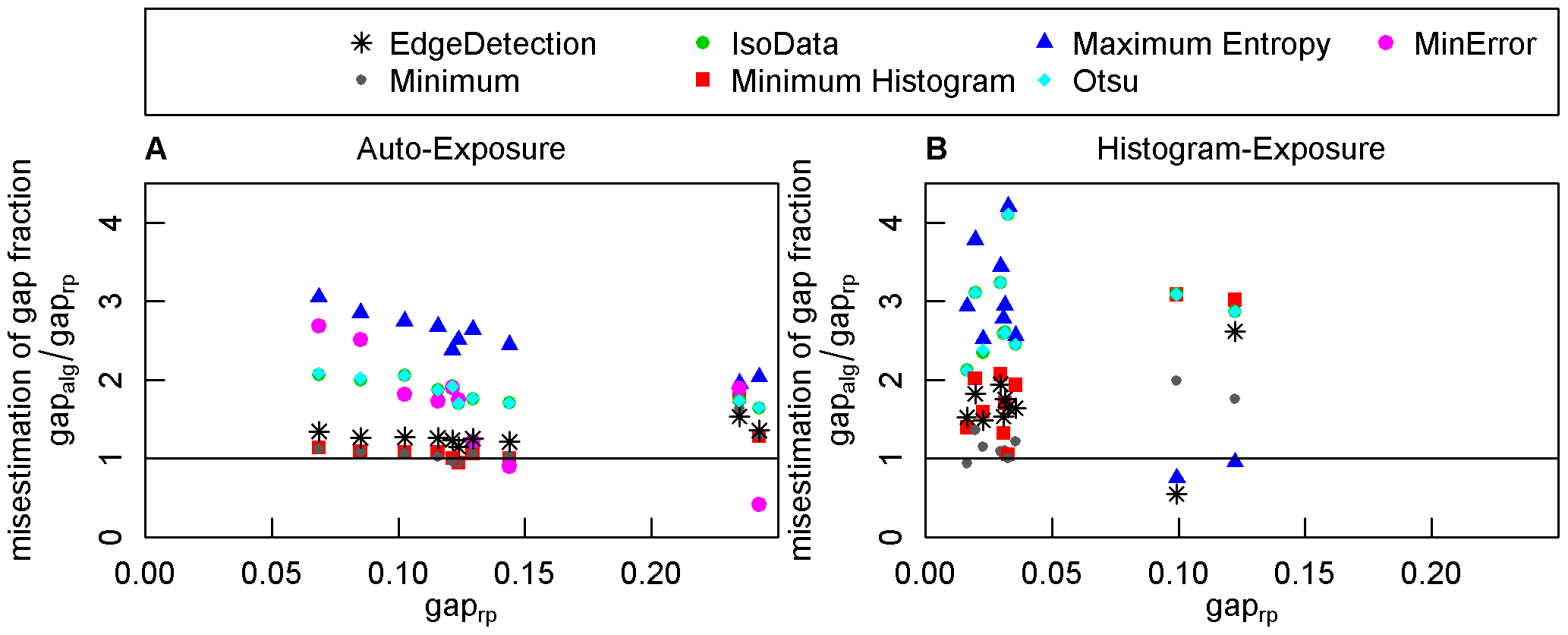
Misestimation of gap fraction values by binarization algorithms. For each algorithm, misestimation was quantified per photograph by dividing the estimated gap fraction of the algorithm (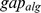) by the gap fraction estimated from manually classified reference pixels (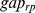). Misestimations are displayed for histogram- and auto-exposed photographs. The horizontal line indicates the best possible classification.

Compared to gap fraction estimates based on the reference pixels, the algorithms of the first group overestimated the gap fraction of the auto-exposed photographs on average by 3% (Minimum), 5% (Minimum-Histogram), and 25% (Edge Detection, Figure 5). Between Minimum and Minimum Histogram there was no significant difference, but the difference of Edge Detection to both algorithms was significant (p-value <0.0000056). The misestimations of the histogram-exposed photographs were with 11%, 63%, and 67% considerably larger. The difference between the Minimum algorithm and both other algorithms of the first group were significant (p-value <0.0052).

All other algorithms overestimated the gap fractions of the auto-exposed photographs by more than 80% and those of the histogram-exposed photographs by more than 180%.

## Discussion

### Accuracy of the algorithms

The algorithms binarized the hemispherical photographs with varying accuracies which in consequence impacted the derived gap fraction estimates. This emphasized the necessity to standardize protocols for the processing of hemispherical photographs.

In terms of accuracy measures the algorithms were ranked similarly for auto-exposed and histogram-exposed photographs. The algorithms Minimum, Minimum Histogram, and Edge Detection were identified to binarize hemispherical photographs with the highest accuracies. The differences between these three algorithms were not significant and also based on the misestimation of gap fraction by these algorithms, a clear assignment of a first rank was not possible. All three algorithms were identified suitable for the classification of hemispherical photographs into sky and vegetation. The occasionally very low and variable accuracies of the other algorithms (IsoData, Maximum Entropy, Otsu, and MinError) indicated that these algorithms should not be used for the binarization of hemispherical photographs.

### The overexposure issue

As auto-exposure leads to a loss of information on sky and of vegetation pixels [4],[8],[9], no algorithm is likely to work correctly with auto-exposed photographs. Even a human operator would not be able to detect vegetation pixels if they were overexposed, hence, in auto-exposed photographs a manual classification of reference pixels is. Therefore, the accuracy measures of the auto-exposed photographs can hardly be interpreted as being calculated on basis of a “true” reference. They should rather be seen as an index which points out the best possible binarizations under the given circumstances.

**Figure 6:**
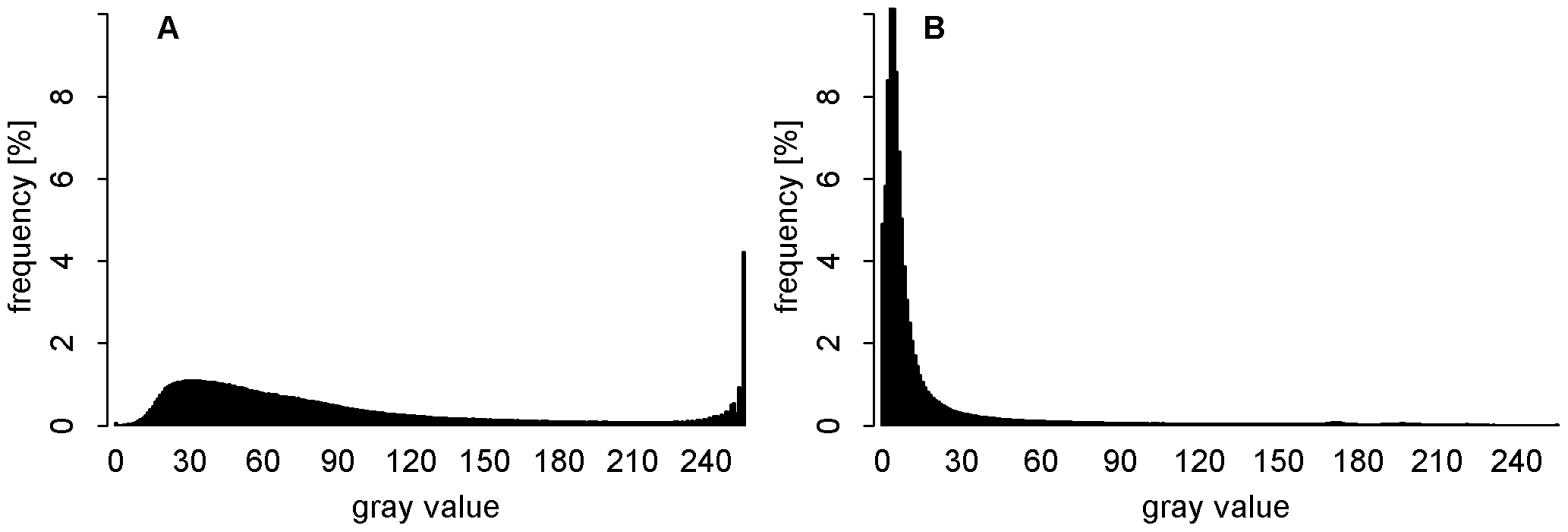
Overexposure of sky regions in hemispherical photographs. Gray value histograms of the blue color plane of the photographs taken on site VI with auto-exposure (A) and histogram-exposure (B).

All tested algorithms except Edge Detection are histogram based and use gray value frequencies for the calculation of thresholds. Overexposure, frequently occurring in auto-exposed photographs, influences the shape of the histogram, with overexposed pixels forming a distinct peak at the bright end of the histogram's x-axis ([5], Figure 6). The shape of a photograph's gray value histogram affects the thresholds set by the algorithms IsoData, Maximum Entropy, and Otsu, which separate vegetation and sky pixels into two distributions. With histogram-exposure the highest gray value is assigned to the brightest spot in the scene. This has the effect that on histogram-exposed photographs a lower threshold is set by the algorithms than on auto-exposed photographs (see section 0). Also the visual impression of the binarized photographs and the overestimation of gap fraction by these algorithms (Figure 5 A and B) indicate that mainly vegetation pixels were misclassified. The estimated binarization accuracies of the histogram-exposed photographs were significantly higher for all three algorithms. Hence, IsoData, Maximum Entropy, and Otsu's method require photographs without overexposed pixels.

The results obtained by the algorithms Minimum and Minimum Histogram might also be affected by overexposure occurring in photographs. Both algorithms search for a gray value with a particular low frequency, i.e. the minimum. As overexposed pixels form a peak at the bright end of the photograph's gray value histogram, the “true” minimum of a scene, separating sky and vegetation, might be concealed in this peak. Thus, both algorithms would not find a scene's global but a local minimum. As gray value frequencies of sky pixels are approximately normally distributed [39] in [46], this might occur if additionally to pure sky pixels vegetation and mixed pixels are overexposed as well. Hence, the algorithms Minimum and Minimum Histogram require hemispherical photographs with no overexposure at all, as e.g. obtained with histogram-exposure, or overexposure limited to sky. With auto-exposed photographs the algorithms Minimum and Minimum-Histogram might not work as intended.

The Edge Detection algorithm is not histogram based; it searches for a threshold which ensures the highest possible local contrast between classes [22]. As the difference between gray values of differently classified, neighboring pixels gets smaller for higher thresholds, a loss of information in very bright parts of photographs has no effect on the threshold determination. Nevertheless, like the algorithms Minimum and Minimum Histogram, Edge Detection requires photographs without overexposure or overexposure limited to gaps, as only in these a scene's “true” threshold is contained.

Some algorithms can handle overexposure if restricted to sky pixels: Edge detection, Minimum, and Minimum Histogram. For these algorithms it might be beneficial to use a slightly higher exposure than determined by the histogram-exposure method. This would result in a higher contrast between vegetation and sky and potentially allow for a better separability of the classes by algorithms and operators alike. Nevertheless, in the field it is hard to assess whether overexposure affects sky pixels only. A possible solution could be to take several photographs with varying exposures at each location, and later on to assess which exposure ensures the highest contrast between vegetation and sky pixels while avoiding overexposing vegetation pixels by displaying the photographs on a large computer monitor.

### Mixed pixels

Mixed pixels are covered by vegetation and sky simultaneously; they cannot be binarized unmistakably by an operator or by an algorithm. Present-day hemispherical photography uses high resolution digital cameras. This decreases the ratio of mixed to non-mixed pixels and negative effects are minimized. For correctly exposed hemispherical photographs this error is small enough to be neglected [13]. Exceptions are photographs like those taken on sites VIII and IX (Figure 1 B) which contain a large amount of mixed pixels because of their high brightness [13] and the high amount of visible fine structures. This does not mean binarization results obtained for such sites are necessarily wrong but a reliable accuracy assessment of classifications of such photographs is difficult.

### Comparison with other studies

[6] proposed the IsoData algorithm as being optimal for the processing of hemispherical photographs. This result was not reproduced by our study. Possible reasons are different exposure settings, another evaluation methodology, and a different way to generate a reference. Also, two of the three best ranking algorithms in our study (Edge Detection and Minimum Histogram) were developed just recently and have not been considered by [6] it remains unknown how they would have performed with the methodology of [6].

[13] concluded that differences between classifications of different algorithms are not substantial and have only little impact on indices obtained from hemispherical photographs. Contrasting, our results suggest that considerable differences in binarization accuracy exist between algorithms. One possible reason for this discrepancy could be that [13] assessed the impact of the algorithms on specific indices, and classification errors which compensate for each other were not accounted for. Another reason for the disagreement of both studies might be the evaluation of different algorithms. None of the algorithms of the present study was addressed and all four algorithms evaluated by [13] work in a similar way in identifying mixed pixels and allocating them evenly to the classes sky and vegetation.

The bias of the Maximum Entropy algorithm towards a lower gray value and a larger gap fraction detected by [14] was also found in our results. The high accuracies of Otsu's method and MinError [14] could not be reproduced by our study. A potential reason for this might be that [14] compared binarizations against interactively thresholded photographs which was identified to be highly subjective by several authors (e.g. [6],[14],[21],[22]).

## Conclusions

The algorithms Minimum, Edge Detection, and Minimum Histogram achieved the highest binarization accuracies. The latter two algorithms misclassified more vegetation pixels and overestimated gap fraction but differences were not statistically significant. All three algorithms were appropriate for the binarization of hemispherical photographs; at this point no recommendation can be given which of them should be preferred.

For the algorithms Maximum Entropy, IsoData, and Otsu's method an incompatibility with auto-exposed photographs containing overexposed pixels was detected. Photographs taken with histogram-exposure were binarized with higher accuracies.

All seven algorithms have been shown to be sensitive to overexposed photographs. Therefore, we strongly recommend applying an exposure determination method which prevents overexposure (e.g. [5],[9]). Besides the investigated binarization algorithms and exposure settings other factors influence the parameters modeled based on hemispherical photographs. For example, gamma correction of pixel gray values applied by digital cameras [21], camera type [47], and light and weather conditions on the site [14] are known to influence results. Until now, studies only dealt with one or two of these factors at a time. A comprehensive study which addresses the influence of all known factors and possible interrelations would be recommended to standardize the acquisition and processing of hemispherical photograph.

## Acknowledgments

We are grateful to all members of the MMC-project (Making the Mekong Connected) for their support. Especially, we thank Mr. Rhett D. Harrison for facilitating the data collection in Xishuangbanna Tropical Botanical Garden (XTBG). We also wish to thank Mr. Collins Boyobona Kukunda for proof-reading the manuscript.
